# Three Binding Conformations of BIO124 in the Pocket of the PICK1 PDZ Domain

**DOI:** 10.3390/cells11152451

**Published:** 2022-08-07

**Authors:** Amy O. Stevens, Samuel Luo, Yi He

**Affiliations:** 1 Department of Chemistry and Chemical Biology, University of New Mexico, Albuquerque, NM 87131, USA; 2 Albuquerque Academy, Albuquerque, NM 87131, USA; 3 Translational Informatics Division, Department of Internal Medicine, University of New Mexico, Albuquerque, NM 87131, USA

**Keywords:** PICK1, PDZ domain, BIO124, dynamic allosterism

## Abstract

The PDZ family has drawn attention as possible drug targets because of the domains’ wide ranges of function and highly conserved binding pockets. The PICK1 PDZ domain has been proposed as a possible drug target because the interactions between the PICK1 PDZ domain and the GluA2 subunit of the AMPA receptor have been shown to progress neurodegenerative diseases. BIO124 has been identified as a sub µM inhibitor of the PICK1–GluA2 interaction. Here, we use all-atom molecular dynamics simulations to reveal the atomic-level interaction pattern between the PICK1 PDZ domain and BIO124. Our simulations reveal three unique binding conformations of BIO124 in the PICK1 PDZ binding pocket, referred to here as state 0, state 1, and state 2. Each conformation is defined by a unique hydrogen bonding network and a unique pattern of hydrophobic interactions between BIO124 and the PICK1 PDZ domain. Interestingly, each conformation of BIO124 results in different dynamic changes to the PICK1 PDZ domain. Unlike states 1 and 2, state 0 induces dynamic coupling between BIO124 and the αA helix. Notably, this dynamic coupling with the αA helix is similar to what has been observed in other PDZ–ligand complexes. Our analysis indicates that the interactions formed between BIO124 and I35 may be the key to inducing dynamic coupling with the αA helix. Lastly, we suspect that the conformational shifts observed in our simulations may affect the stability and thus the overall effectiveness of BIO124. We propose that a physically larger inhibitor may be necessary to ensure sufficient interactions that permit stable binding between a drug and the PICK1 PDZ domain.

## 1. Introduction

**Figure 1:**
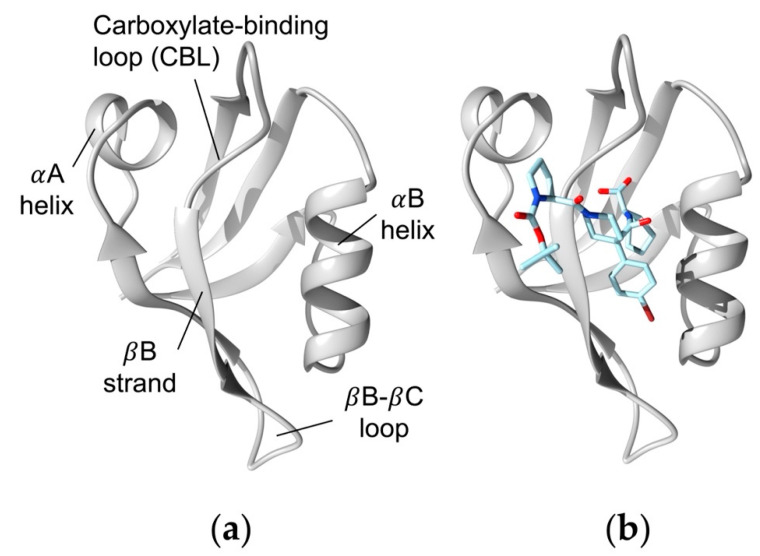
The PICK1 PDZ domain. (**a**) PICK1 PDZ domain with labeled secondary structures (PDB ID: 6BJO). (**b**) PICK1 PDZ-BIO124 complex with BIO124 shown in blue (PDB ID: 6BJO). Note that (**b)** is the starting structure of the all-atom MD simulations.

The PDZ (PSD-95/Dlg1/ZO-1) family is a large protein family that is involved in protein–protein interactions and regulating signaling pathways [1,2,3,4,5,6]. Over 250 PDZ domains have been identified in more than 100 human proteins [7]. These domains have been shown to have key roles in various biological processes, including managing cell polarity, regulating tissue growth, trafficking membrane proteins, and regulating cellular pathways [8,9,10]. The PDZ family has highly conserved secondary structure, as shown in Figure 1a. Most commonly, the PDZ domain forms protein–protein interactions with the final three to five C-terminal residues of target proteins at the conserved PDZ binding pocket [11]. The PDZ binding pocket is a hydrophobic groove between the αB helix and the βB strand [12]. Together, the sheer abundance of PDZ domains, their wide range of function, and their highly conserved binding pattern has drawn attention to PDZ domains as possible drug targets [13,14,15]. For example, many inhibitors have been designed to target the PDZ domains of SHANK genes [16,17], Dishelved proteins [18,19,20,21,22,23,24], Scribble [25], Syntenin [26,27], PSD-95 [28,29,30,31] and PICK1 [32,33,34,35].

PICK1 (Protein Interacting with C Kinase-1) is an especially unique PDZ-protein as it is the only protein in the human proteome that contains both a PDZ domain and a BAR (Bin/amphiphysin/Rvs) domain [36,37,38]. This combination of modular domains allows PICK1 to have unique biological functions. PICK1 is found in multiple tissues and organs where it is involved in regulating the trafficking of various membrane proteins [39,40,41], including the Dopamine Transporter [42] and the AMPA receptor [39]. Because of its functional role in regulating various neurotransporter receptors, transporters, and enzymes, PICK1 has been suggested to play a role in neurological diseases, such as chronic pain, epilepsy, stroke, Parkinson’s disease, schizophrenia, and substance use disorders [43]. Researchers have sought to identify inhibitors to target PICK1 and be used as potential therapeutics. FSC231 was the first-identified small-molecule inhibitor of PICK1 [44]. FSC231 showed efficacy in reducing the expression of both long-term depression and long-term potentiation in hippocampal cornu ammonis 1 (CA1) neurons from acute slices, signifying the inhibition of PICK. In addition to FSC231, peptide inhibitors such as Pep2-EVKI and Pep2-SVKI have also proved to be effective at targeting the PICK1 PDZ domain [35]. Specifically, Pep2-EVKI was shown to reduce cocaine seeking in mice [45]. More recently, the bivalent peptide inhibitor TAT-P_4_-(C5)_2_ was shown to alleviate neuropathic pain [46,47]. While the various efforts described above have all sought to target the PICK1 PDZ domain, here we will focus on the small-molecule inhibitor BIO124 (Figure S1).

BIO124 is a sub µM inhibitor of the PICK1–GluA2 interaction that was identified by Marcotte et al. using a fluorescence polarization assay [32]. The PICK1–GluA2 interaction is responsible for the trafficking of the AMPA receptor away from the neuron surface [48,49]. As AMPA receptors are primarily localized on the surface of neurons, they play a key role in mediating synaptic communication [50,51]. It has been shown that the down regulation of AMPA receptors can lead to progression in neurodegenerative diseases such as Alzheimer disease, thus suggesting that inhibiting the PICK1-GluA2 interaction that is responsible for such down regulation may be an effective therapy for Alzheimer disease [52]. With this goal in mind, Marcotte et al. identified BIO124 [32]. BIO124 displayed a half-maximal inhibitory concentration (IC_50_) of 0.51 µM, signifying good activity. Marcotte et al. produced a co-crystal structure of the PICK1 PDZ-BIO124 complex that revealed an interaction pattern that mimics the interactions with natural ligand GluA2.

The purpose of this study is to use all-atom molecular dynamics (MD) simulations to explore how the atomic-level interaction pattern between the effective sub-µM inhibitor BIO124 and the PICK1 PDZ domain affects the dynamics of the PICK1 PDZ domain. Because the PDZ family is a classic example of dynamic allostery in small modular domains, it has been of particular interest to explore how natural ligands trigger dynamic changes in various PDZ domains. We will apply MD simulations to explore how BIO124, which was designed to mimic natural ligand GluA2, alters the dynamics of the PICK1 PDZ domain. MD simulations have proved to be a reliable tool to explore the dynamics of the PDZ family [53,54,55,56,57]. The system of interest is shown in Figure 1b. We see that BIO124 has three unique binding conformations within the PICK1 PDZ binding pocket that we refer to as state 0, state 1, and state 2. Each binding conformation is defined by a unique hydrogen bonding network and hydrophobic interaction pattern between the inhibitor and the PICK1 PDZ domain. Interestingly, our results indicate that each interaction pattern between BIO124 and the PICK1 PDZ domain can induce unique dynamic changes to the PICK1 PDZ domain. Specifically, state 0 of BIO124 directly affects the dynamics of the αA helix of the PICK1 PDZ domain, a site that is distal from the binding pocket. We suspect that interactions between BIO124 and I35 may be a key player in this process.

## 2. Materials and Methods

We modeled the PICK1 PDZ–BIO124 complex with all-atom MD simulations. BIO124 is a small-molecule inhibitor designed by Marcotte et al. to mimic PICK1 PDZ-GluA2 interactions [32]. The experimentally determined crystal structure of the complex was used to generate the starting structure for all simulations (PDB ID: 6BJO [32]). To allow direct comparison to our future work, the PDB file was manually edited by trimming terminal residues to ensure an identical sequence to the PICK1 PDZ-GluR2 system. The starting structure is shown in Figure 1b. The system was prepared using CHARMM-GUI [58,59]. The most recently developed CHARMM36m [60] force field with explicit solvent (TIP3P) was used with the Groningen Machine for Chemical Simulations (GROMACS) package [61,62,63] version 2020.4. Counter ions (Na^+^ or Cl^−^) were added to neutralize the system at 293 K. Steepest-descent minimization and a 1 ns MD equilibrium simulation was carried out to generate equilibrated starting structures for the MD simulations. All bonds with hydrogen atoms were converted to constraints with the algorithm LINear Constraint Solver (LINCS) [64], and a Nose-Hoover temperature thermostat [65,66] was used. The time step was set as 2 fs, and snapshots were taken every 100 ps. The system was built in a 90 Å × 90 Å × 90 Å cubic water box. We performed four replicates of a 7 µs trajectory, a total of 28 µs (4 × 7 µs) of simulations.

We calculated the distance between binding pocket residue I37 and BIO124 to monitor the possibility of any dissociation events during the simulations. Distance plots are shown in Figure S2. The distance between I37 and BIO124 suggests that BIO124 remained in the binding pocket during trajectories 1, 2, and 3. During trajectory 4, the distance between I37 and BIO124 sharply increases after ~4 µs, signifying the dissociation of BIO124 from the binding pocket. Cluster analysis was used to confirm this dissociation. The top ten clusters of BIO124 during each trajectory are shown in Figure S3. As shown in Figure S3, all frames of trajectories 1, 2, and 3 fit into clusters that place BIO124 in the binding pocket of the PICK1 PDZ domain. Oppositely, trajectory 4 reveals clusters with BIO124 having dissociated from the binding pocket. Lastly, we performed two sets of cluster analysis over trajectory 4. We performed cluster analysis across the first 4 µs of trajectory 4 and the final 3 µs of trajectory 4, as shown in Figure S4a,b, respectively. The results confirm that, during the first 4 µs of trajectory 4, BIO124 remains in the binding pocket. With this, all further analysis will be performed over all frames of trajectories 1, 2, and 3 and only the first 4 µs of trajectory 4.

Protein network analysis was used to describe the allosteric network between BIO124 and the PICK1 PDZ domain. Protein network analysis calculates the correlated movements between residues within a protein or protein complex by constructing residue-based and community-based weighted network graphs based on a trajectory. Each residue is represented by a node in a network and the links between nodes are the cross-correlation values between these nodes. The displacement of the Cα atoms are used to assess the magnitude of all pairwise cross-correlation coefficients [67]. Correlation coefficients range from −1 to 1. A value of 1 indicates that the fluctuations of two Cα atoms are completely correlated, a value of 0 indicates that the fluctuations of two Cα atoms are not correlated, and a value of −1 indicates that the fluctuations of two Cα atoms are completely anticorrelated (same period and opposite phase). The correlation coefficients return a community partition with the highest overall modularity value based on Girvan-Newman style clustering [68]. Analysis was carried out using the bio3d package [69,70,71].

Time-resolved force distribution analysis (TRFDA) [72] was used to reveal the punctual stress on each PICK1 PDZ residue as a result of interactions with BIO124. TRFDA traces the changing force on atoms/residues of interest that results from a perturbation. In our case, the perturbation is the binding of BIO124. The calculated changing forces are transformed into punctual stresses per residue. TRFDA was carried out to obtain the punctual stresses on PICK1 PDZ residues as a result of perturbations from interactions with BIO124.

## 3. Results

**Figure 2:**
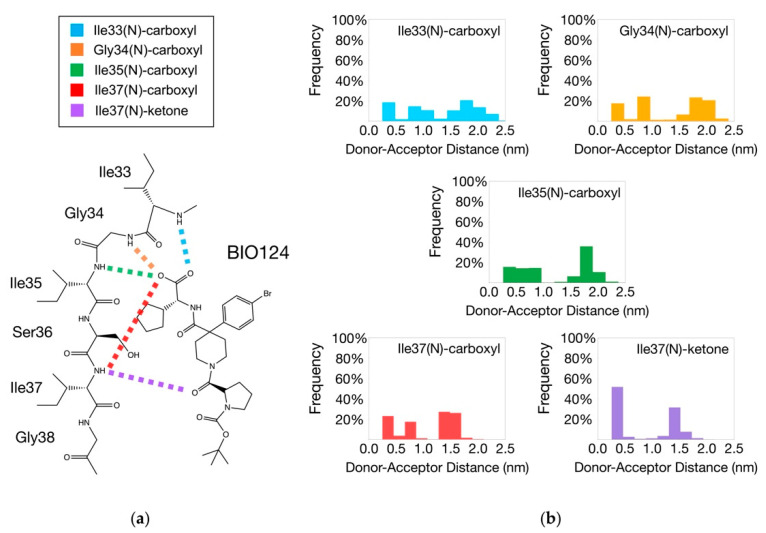
Hydrogen bonding network between BIO124 and the PICK PDZ domain. (**a**) Hydrogen bonds identified in both experimental work and our MD simulations include I33-carboxyl (cyan), G34-carboxyl (orange), and I35-carboxyl (green). Hydrogen bonds identified only in our MD simulations include I37-carboxyl (red) and I37-ketone (purple). (**b**) Distance distribution between each hydrogen bonding pair in the complex. Note that each hydrogen bonding pair produces distinct distance peaks.

Hydrogen bond analysis was performed to explore the interaction pattern between BIO124 and the PICK1 PDZ domain in our simulations and to serve as a direct comparison to experimental results. The analysis revealed an interaction pattern that is in relatively good agreement with the crystal structure of the PICK1 PDZ-BIO124 complex (PDB ID: 6BJO [32], Figure 2a). The crystal structure of the complex revealed that the carboxylic acid of BIO124 forms hydrogen bonds with the backbone of I33, G34, and I35 [32]. Our simulations also identified the presence of these three hydrogen bonds. Figure 2a displays the I33–carboxyl, G34-carboxyl, and I35-carboxyl hydrogen bonds in cyan, orange, and green, respectively. Interestingly, our simulations produced the formation of two additional hydrogen bonds, including the carboxylic acid of BIO124 with the backbone of I37 and the center ketone of BIO124 with the backbone of I37. Figure 2a displays the I37-carboxyl and I37-ketone hydrogen bonds in red and purple, respectively. Next, we performed a statistical analysis to rank the probability of each hydrogen bond forming in the binding pocket (Figure 2b). If BIO124 took a singular conformation for all combined frames of the MD simulations, we would expect the distance between each hydrogen bonding pair to fluctuate around one value and ultimately produce a Gaussian distribution. Surprisingly, the distance distributions between each hydrogen bonding pair reveal a non-Gaussian distribution. The distinct peaks observed in Figure 2b suggest the presence of multiple binding conformations between BIO124 and the PICK1 PDZ domain.

**Figure 3:**
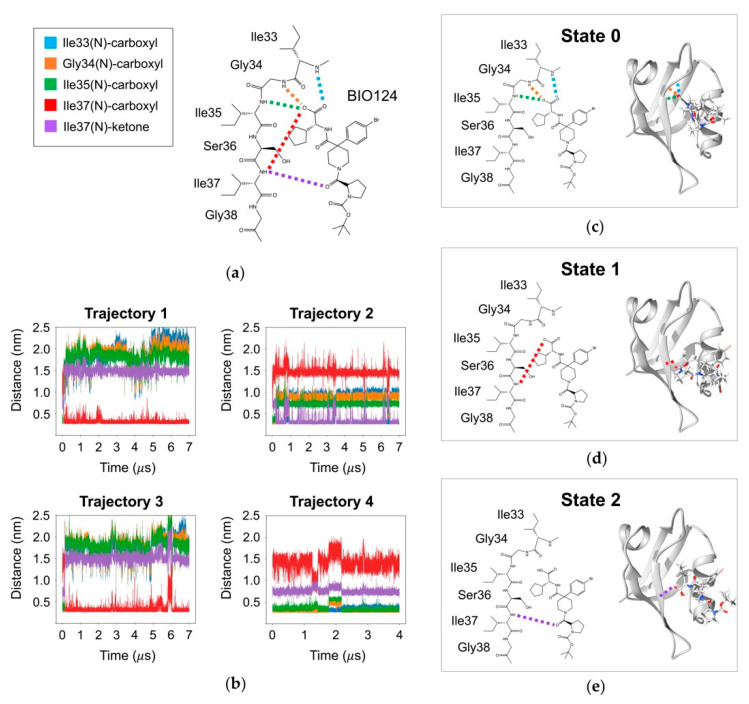
The three binding conformations of BIO124. (**a**) Hydrogen bonding pairs between BIO124 and the PICK1 PDZ domain. (**b**) Atom-atom distance analysis of the hydrogen bonding pairs in the PICK1 PDZ-BIO124 complex by trajectory. Distance analysis suggests unique binding conformations of BIO124. (**c**) State 0 of BIO124 (trajectory 4). Hydrogen bonding network (left) and cluster analysis (right) are in good agreement. State 0 resembles the conformation of the ligand in the crystalized structure. (**d**) State 1 of BIO124 (trajectories 1 and 3). Hydrogen bonding network (left) and cluster analysis (right) are in good agreement. State 1 is new stable conformations taken by the ligand during our simulations. (**e**) State 2 of BIO124 (trajectory 2). Hydrogen bonding network (left) and cluster analysis (right) are in good agreement. State 2 is new stable conformations taken by the ligand during our simulations.

We explore the possibility of multiple binding conformations between BIO124 and the PICK1 PDZ domain by calculating the atom-atom distances between each hydrogen bonding pair (Figure 3a). Atom-atom distance analysis (Figure 3b) was performed over each hydrogen bonding pair in the PICK1 PDZ–BIO124 complex (I33-carboxyl, G34-carboxyl, I35-carboxyl, I37-carboxyl, and I37-ketone). The distance curve for each pair is specified by color in Figure 3a. Atom-atom distance analysis can be used to track the breaking and forming of hydrogen bonding pairs during the simulations. Here, we correlate these distance changes to the breaking of hydrogen bonds if the distance between the atoms involved in the hydrogen bond is greater than 0.5 nm. Assuming a unique pattern of hydrogen bonds corresponds to a unique binding conformation, our simulations suggest that three unique binding conformations exist between BIO124 and the PICK1 PDZ domain. Trajectory 4 keeps the conformation of the starting structure with three hydrogen bonds formed throughout the trajectory, including I33–carboxyl, G34-carboxyl, and I35-carboxyl. During trajectories 1 and 3, the initial hydrogen bonds (I33-carboxyl, G34-carboxyl, I35-carboxyl) are almost immediately broken while a new hydrogen bond between the backbone of I37 and the center ketone of BIO124 is formed. During trajectory 2, the same initial hydrogen bonds are broken while a new hydrogen bond between the backbone of I37 and the carboxylic acid of BIO124 is formed. Our results suggest that BIO124 can make an initial conformational change and then remain in the new stable state for the remainder of the trajectory.

Assuming that the unique sets of hydrogen bonds formed in each trajectory point to unique binding conformations of BIO124, we repeated the statistical analysis of the hydrogen bonding networks based on each conformation. We will refer to the frames in trajectory 4 as state 0, the frames in trajectories 1 and 3 as state 1, and the frames in trajectory 2 as state 2. First, hydrogen bond analysis was performed over each state. Hydrogen bond analysis identified the presence of three hydrogen bonding pairs in state 0, including I33-carboxyl, G34-carboxyl, and I35-carboxyl (Figure S5a). Subsequently, hydrogen bond analysis identified the presence of the I37-ketone pair and the I37-carboxyl pair in state 1 and state 2, respectively (Figures S6a and S7a). Next, we performed a statistical analysis to rank the probability of each hydrogen bond forming in each state (Figures S5b, S6b and S7b). By separating the frames into states, the distance distributions between each hydrogen bonding observe a Gaussian distribution, signifying a stable binding conformation within each state.

Lastly, the presence of unique binding conformations between BIO124 and the PICK1 PDZ domain was confirmed with cluster analysis. Suspecting three unique binding conformations, we divided the combined PICK1 PDZ-BIO124 trajectories into three clusters. The results agree with our suspicions drawn from hydrogen bond analysis. As shown in Figure 3c–e, cluster analysis produced three unique binding conformations that correspond to the three unique hydrogen bond networks. State 0 resembles the conformation in the crystal structure (PDB ID: 6BJO [32]) with the presence of three hydrogen bonds, including I33-carboxyl, G34-carboxyl, and I35-carboxyl. In state 1, BIO124 flips upside down so that its center ketone forms a hydrogen bond with the backbone of I37. In state 2, BIO124 shifts down in the binding pocket so that its carboxylic acid forms a hydrogen bond with the backbone of I37. The size of each cluster is in relatively good agreement with the ratio of frames with each corresponding atom-atom distance pair. The cluster corresponding to state 0 represents 31.7% of the total frames, the cluster corresponding to state 1 represents 46.2% of the total frames, and the cluster corresponding to state 2 represents 22.1% of the total frames.

The analyses described above point to three unique bonding conformations between BIO124 and the PICK1 PDZ domain. Only one of these conformations (state 0) corresponds to the X-ray structure of the PICK1 PDZ–BIO124 complex (PDB ID: 6BJO) [32]. The X-ray structure of the PICK1 PDZ–BIO124 complex was crystalized at 4 °C [32]. Together, the low temperature and the space restraints inherently induced during crystallization can limit the sampling of conformational states. MD simulations in this work were performed at room temperature (293 K or ~20 °C). This increase in temperature may permit the sampling of states that is readily observed in this work. To explore this possibility, additional MD simulations were performed at 277 K (~4 °C), which corresponds to the temperature used in the PDZ-BIO124 X-ray experiments. At 277 K, the conformation of BIO124 is in good agreement with the X-ray structure of the PICK1 PDZ-BIO124 complex, and no conformational shifts to reach states 1 and 2 were observed. The additional states observed in the 293 K simulations may be a result of an increase in temperature that permits the sampling of states. These results are included in the SI. Figures S8 and S9 shows the hydrogen bonding network and the atom-atom distance analysis at 277 K, respectively.

**Figure 4:**
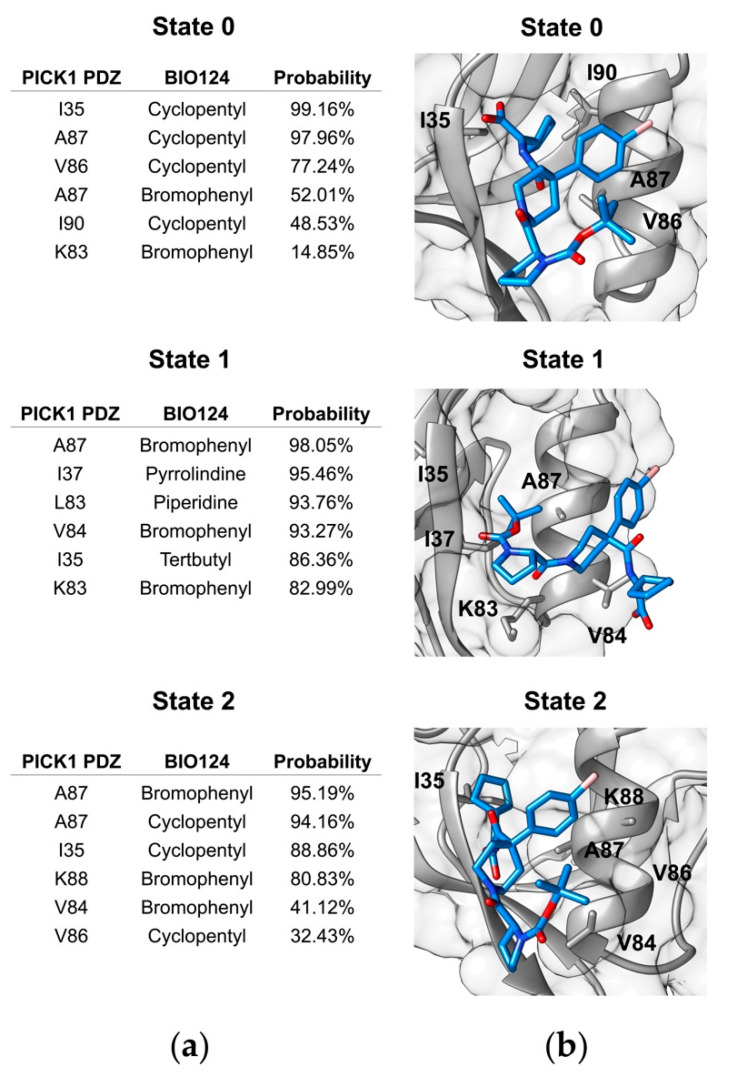
Hydrophobic interactions between BIO124 and the PICK1 PDZ domain across the three states of BIO124. (**a**) Most probable contacts between BIO124 and the PICK PDZ domain. (**b**) Visual representation of hydrophobic interactions between BIO124 and the PICK PDZ domain.

Our simulations produce three binding conformations of BIO124 that correspond to three unique hydrogen bonding networks. While hydrogen bonds are one category of key atomic-level interactions in protein-ligand binding, we also wanted to explore the role of hydrophobic forces in each of the three states of BIO124. The crystal structure of the PICK1 PDZ-BIO124 complex reveals various hydrophobic interactions. In the crystal structure, the cyclopentyl group of BIO124 forms hydrophobic interactions with I37, A87, and I90. The piperidine core of BIO124 forms hydrophobic interactions with L32 and F53. Lastly, the bromophenyl moiety aligns with the carbon chain of K83. To explore the hydrophobic interactions in our simulations, we performed contact map analysis across each state of BIO124. We only consider the contact formed with heavy atoms of BIO124. The most probable contacts with the complex at each state are listed in Figure 4a, and visual representations of each state are shown in Figure 4b. In state 0, the cyclopentyl group of BIO124 forms frequent contact with I35 (98.90%), A87 (97.93%), V86 (73.26%), and I90 (54.55%). Additionally, the bromophenyl moiety forms contact with the carbon chain of K83 (16.49%). Hydrogen bond analysis and cluster analysis suggests that State 0 of BIO124 most readily resembles the conformation of BIO124 in the crystal structure of the PICK1 PDZ-BIO124 complex. Contact map analysis echoes these results as our simulations point to key hydrophobic interactions between (1) the cyclopentyl group and I35, A87, and I90 and (2) the bromophenyl moiety and the carbon chain of K83. Interestingly, our simulations fail to reproduce the frequent contact between the piperidine core and L32/F53 that was observed in the crystal structure. In state 1 and state 2, BIO124 has taken new conformations in the PICK1 PDZ binding pocket and thus has adopted novel hydrophobic interaction patterns. For example, in state 1, the bromophenyl moiety of BIO124 forms frequent contact with A87 (96.05%), V84 (93.44%), and L83 (85.18%). Additionally, the pyrrolindine moiety frequently contacts I37 (94.16%), the piperidine core frequently contacts L83 (87.05%), and the tertbutyl group frequently contacts I35 (83.59%). In state 2, the cyclopentyl group forms frequent contact with I35 (98.00%), A87 (89.75%), and V86 (39.47%). The hydrophobic interactions between the cyclopentyl group and I35, A87, and V86 closely resembles those observed in state 0. Unique from state 0, the bromophenyl group forms much more frequent contact with A87 (91.31%) in state 2. Furthermore, in state 2, the electronegative carboxyl group forms a charged interactions with the electropositive ammonium in K83.

While states 1 and 2 are not observed in the experimental structure of the PICK1 PDZ-BIO124 complex, many of the interaction patterns observed in these states are characteristic of the atomic-level interactions between the PICK1 PDZ domain and natural ligand GluR2 (PDB ID: 2PKU) [73], for which BIO124 is a mimic. (Notably, the final five C-terminal residues of GluR2 and GluA2 are identical.) For example, the experimentally determined structure of the PICK1 PDZ-GluR2 complex points to the importance of the hydrophobic interaction between the side chain of Val(-2) of GluR2 and K83/A87 of the αB helix of the PICK1 PDZ domain. A similar interaction is observed in state 1 of our simulations where the bromophenyl moiety is similarly sandwiched between A87 (98.05%) and K83 (82.99%). Furthermore, the experimentally determined structure reveals a charge-charge interaction between the side chains of Glu(-4) of GluR2 and K83 of the PICK1 PDZ domain. In state 2 of our simulations, we observed a similar charge-charge interaction between the electronegative carboxyl group of BIO124 and the electropositive ammonium of K83 of the PICK1 PDZ domain. This suggests that while states 1 and 2 are not observed in the X-ray structure of the PICK1 PDZ-BIO124 complex, the atomic-level interactions observed in states 1 and 2 are characteristic of critical binding patterns between the PICK1 PDZ domain and its natural ligand.

Time-resolved force distribution analysis (TRFDA) was performed to reveal the punctual stress on each PICK1 PDZ residue as a result of interactions with BIO124 [72]. TRFDA can expose which residues are key in holding the ligand in place. The analysis was performed over each trajectory and the per-trajectory results were subsequently summed. The summed results are shown in Figure S10. The ten PICK1 PDZ residues that experienced the greatest punctual stress for each state are listed in Figure S11. In state 0, BIO124 induces the greatest punctual stress on residues composing the βB strand. Oppositely, in state 1 and state 2, BIO124 induces the greatest punctual stress on residues composing the αB helix. These results further point to key differences in the binding mechanisms between BIO124 and the PICK1 PDZ domain in each conformational state of BIO124.

**Figure 5:**
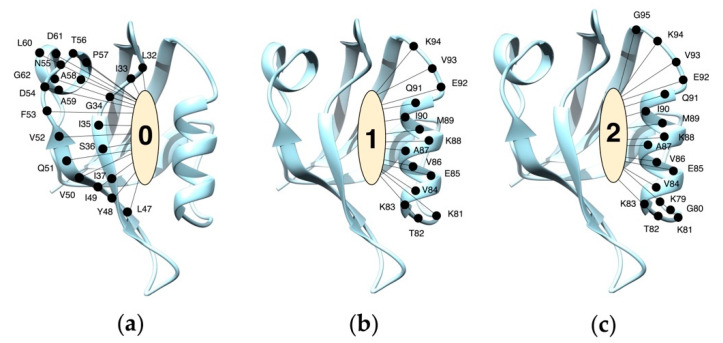
Protein network analysis across the three states of BIO124. (**a**) In State 0, the major motions of BIO124 couple with the βB strand, βC strand, and αA helix of the PICK1 PDZ domain. (**b**) In State 1, the major motions of BIO124 couple with the αB helix of the PICK1 PDZ domain. (**c**) In State 2, the major motions of BIO124 couple with the αB helix of the PICK1 PDZ domain.

The analyses described above reveal that the three binding conformations of BIO124 correspond to three unique sets of atomic-level interactions between BIO124 and the PICK1 PDZ domain. We suspect that each set of interactions will uniquely affect the dynamics of the PICK1 PDZ domain. To explore this possibility, we performed protein network analysis to reveal the coupling of major movements by creating protein structure networks based off the primary motions of each residue. As shown in Figure 5, protein structure network analysis reveals that the unique interaction pattern of each state of BIO124 alters the coupling of major movements within the PICK1 PDZ-BIO124 complex. In state 0, the major motions of BIO124 are coupled to the βB strand, βC strand, and αA helix of the PICK1 PDZ domain (Figure 5a). Oppositely, in states 1 and 2, the major motions of BIO124 are coupled to the αB helix of the PICK1 PDZ domain (Figure 5b,c).

Protein network analysis reveals that the unique conformational states of BIO124 can induce unique dynamic changes to the PICK1 PDZ domain. We suspect that these dynamical differences are in direct relation to the atomic-level interaction pattern between BIO124 and the PICK1 PDZ domain. This begets the question—what specific atomic-level interactions between BIO124 and the PICK1 PDZ domain are responsible for the unique dynamic changes in each binding conformation? Here, we will specifically address the dynamic coupling between BIO124 and αA helix that occurs in state 0.

Our recent review of allosterism in the PDZ family [74] revealed that the αA helix has been consistently identified as an allosteric region by various experimental and computational techniques [54,56,57,75,76,77,78,79,80,81,82]. Furthermore, previous work has identified pathways that may be responsible for the propagation of signal from the ligand to the αA helix through key residues on the βB strand [78,83,84]. For example, in the PTP-BL PDZ2 domain, signal propagates from the ligand to I20 (βB strand) and finally to A46 (αA helix) [84]. In the PSD-95 PDZ3 domain, signal propagates from the ligand to F325 (βB strand) and finally to A347 (αA helix) [78]. The structural alignment of PICK1 PDZ, PTP-BL PDZ2, and PSD-95 PDZ3 (Figure S12) suggests that the allosteric alanine residue (A46/A347) on the αA helix is evolutionarily conserved across all three PDZ domains. The structural equivalents of A46/A347 and I20/F325 on the PICK1 PDZ domain are A58 and I35, respectively. We suspect that interactions between the ligand and I35 of the PICK1 PDZ domain may have a role in the propagation of signal to the αA helix. The three binding conformations of BIO124 present a unique opportunity to explore our hypothesis.

**Figure 6:**
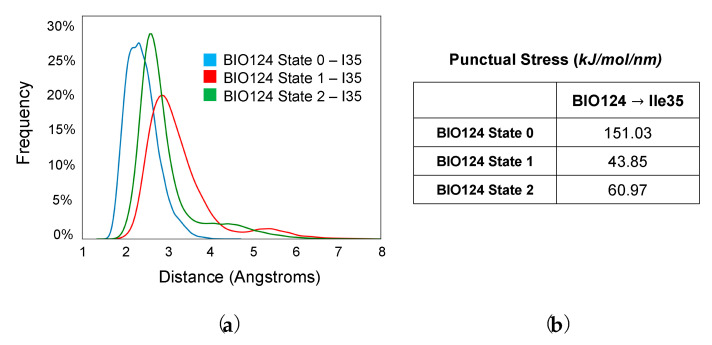
The role of I35 in inducing dynamic allosterism at the αA helix of the PICK PDZ domain. (**a**) Distance distribution between I35 of the PICK1 PDZ domain and BIO124 in each state. (**b**) Punctual stress on I35 of the PICK1 PDZ domain induced by BIO124 in each state.

In effort to explore the role of I35 in propagating signal to the αA helix, we performed distance distribution analysis and time-resolved force distribution analysis (TRFDA) to gain an in-depth understanding of the interactions between BIO124 and I35 in the three conformational states of BIO124. As shown in Figure 6a, distance distribution analysis was performed between BIO124 and I35 for each state. Here, distance is defined as the shortest distance between any two atoms in BIO124 and I35. BIO124 forms the closest contact with I35 in state 0 (blue). Next, we calculated the punctual stress on I35 induced by the BIO124 by using TRFDA. As shown in Figure 6b, BIO124 induces the greatest punctual stress on I35 in state 0. Together, distance distribution analysis and TRFDA reveal that the conformation of BIO124 in state 0 forms the closest contact with and induces the greatest punctual stress on I35. Interestingly, BIO124 only affects the dynamics of the αA helix in state 0. These results point to the importance of interactions between the ligand and I35 in inducing dynamic allosterism at the αA helix of the PICK1 PDZ domain. It is also important to note that, compared to state 1 and state 2, the interactions with I35 in state 0 most closely resemble those with the natural ligand GluR2 [73].

## 4. Discussion

The purpose of this work is to use all-atom MD simulations to investigate how the atomic-level interactions between BIO124 and the PICK1 PDZ domain affect the dynamics of the PICK1 PDZ domain. We found that (1) BIO124 has multiple binding conformations with the PICK1 PDZ domain, (2) the three unique binding conformations of BIO124 result in unique dynamic changes to the PICK1 PDZ domain, and (3) interactions between BIO124 and I35 may be key to inducing dynamic allosterism at the αA helix.

Our simulations reveal three unique binding conformations between the PICK1 PDZ domain and BIO124, referred to here as state 0, state 1, and state 2. Each conformation is characterized by a unique hydrogen bonding network. In state 0, BIO124 forms a hydrogen bonding network with the backbone of I33, G34, and I35. These hydrogen bonds agree with those observed in the experimental structure of the PICK1 PDZ–BIO124 complex [32]. In state 1 and state 2, different regions of BIO124 form a hydrogen bond with the backbone of I37. Interestingly, while hydrogen bonding with I37 is not observed in the experimental structure of the PICK1 PDZ-BIO124 complex, it is characteristic of interactions between the PICK1 PDZ domain and natural ligands [73,85]. In addition to unique hydrogen bonding networks, each conformation of BIO124 is characterized by a unique set of hydrophobic interactions with the PICK1 PDZ domain. In state 0, the cyclopentyl group of BIO124 forms frequent contact with I35, V86, A87, and I90. In state 1, the bromophenyl moiety of BIO124 forms frequent contact with L83, L84, and A87. Additionally, the pyrrolindine moiety, the piperidine core, and the tertbutyl group frequently contact I37, L83, and I35, respectively. In state 2, the cyclopentyl group forms frequent contacts with I35, V86, and A87 that closely resemble those observed in state 0. Unique from state 0, the electronegative carboxyl group of BIO124 forms a charged interaction with the electropositive ammonium in K83.

While the three binding conformations of BIO124 may indeed be an artifact of our simulations, they present a unique opportunity to explore the role of specific interactions in inducing specific dynamic changes to the PICK1 PDZ domain. Our results indicate that the conformation of BIO124 directly affects the dynamics of the PICK1 PDZ domain. In state 0, the major motions of BIO124 are coupled with the carboxylate-binding loop, βB strand, βC strand, and αA helix of the PICK1 PDZ domain. In state 1 and state 2, the major motions of BIO124 are coupled with the αB helix of the PICK1 PDZ domain. It is worth stressing that state 0 of BIO124 can induce dynamic changes to regions of the PICK1 PDZ domain that are distal from the binding pocket, including the βC strand and the αA helix. Previous work has identified the αA helix of the PDZ family as a region affected by dynamic allostery and has pointed to a pathway of signal transduction from structural equivalents of I35 to structural equivalents of A58 [78,83,84]. Our results also support the role of interactions between the ligand and I35 as a key player in propagating signal to the αA helix. In state 0, BIO124 forms both the closest contact with and induces the greatest punctual stress on I35. These results suggest that the interaction pattern between state 0 of BIO124 and the PICK1 PDZ domain provides the necessary signals to induce dynamic allostery at the αA helix of the PICK1 PDZ domain.

Inhibiting PICK1–GluA2 interactions may be an effective therapy for Alzheimer disease [52] and, potentially, could address substance use disorders as well. Previous efforts have identified sub µM inhibitors that demonstrate efficacy in targeting the PICK1 PDZ domain and disrupting interactions with GluA2, including BIO922 [52] and BIO124 [32]. The crystal structure of the PICK1 PDZ-BIO124 complex reveals an interaction pattern that mimics the interactions with the natural ligand GluA2. Here, our simulations reveal additional conformations of BIO124 in the PICK1 PDZ binding pocket (states 1 and 2). While the atomic-level interactions in states 1 and 2 are dissimilar to those observed in the experimental structure of PICK1 PDZ-BIO124, the novel conformations remain characteristic of interactions with the natural ligand [73]. For example, in states 1 and 2, BIO124 forms a hydrogen bond with I37, a hydrophobic core with K83/A87, and a charge-charge interaction with K83. Furthermore, additional simulations performed at 277 K suggest that the population of states 1 and 2 may be a result of an increase in temperature that permits the sampling of states. Point mutation experiments and nuclear magnetic resonance (NMR) experiments performed at room temperature may be useful approaches to explore these novel conformations.

Each conformation of BIO124 dynamically couples with different regions of the PICK1 PDZ domain to form a stable complex. For example, in state 0, BIO124 dynamically couples with the βB strand, βC strand, and αA helix, and in states 1 and 2, BIO124 dynamically couples with the αB helix. Interestingly, natural ligands binding to well-studied PDZ domains such as PSD-95 PDZ3 and PTP-BL PDZ2 have been shown to induce allosteric networks that include residues from both regions described above [54,55,56,57,78,80,81,82,86,87]. While each state of BIO124 emulates the interactions of the natural ligand to some degree, the conformational shifts of BIO124 between such states that were observed in our simulations may affect the stability and thus the overall effectiveness of the drug. We propose that a physically larger inhibitor may be necessary to ensure sufficient interactions that permit stable binding between a drug and the PICK1 PDZ domain. A larger inhibitor may be able to encompass all the necessary atomic-level interactions to emulate both dynamic coupling patterns and thus increase the binding affinity overall. Lastly, the novel conformations observed in our simulations fail to induce dynamic allostery at the distal βC strand and the αA helix as the natural ligand does. Our results suggest that an inhibitor may not need to mimic natural ligand interactions to form stable interactions with the target protein. These results suggest a new potential strategy in drug development that would widen the doors for possible inhibitors.

## Data Availability

All the simulation data can be downloaded at: https://tinyurl.com/47zk92xt (accessed on 3 August 2022).

## Supplementary Materials

The following supporting information can be downloaded at: https://www.mdpi.com/article/10.3390/cells11152451/s1, Figure S1: Structure of BIO124; Figure S2: Distance between I37 of the PICK1 PDZ domain and BIO124; Figure S3: Cluster analysis of BIO124 per trajectory; Figure S4: Cluster analysis of BIO124 in trajectory 4; Figure S5: State 0 hydrogen bonding network between BIO124 and the PICK1 PDZ domain; Figure S6: State 1 hydrogen bonding network between BIO124 and the PICK1 PDZ domain; Figure S7: State 2 hydrogen bonding network between BIO124 and the PICK1 PDZ domain; Figure S8: 277 K hydrogen bonding network; Figure S9: 277 K atom-atom distance analysis of the hydrogen bonding pairs; Figure S10: Summed time-resolved force distribution analysis (TRFDA) for each state of BIO124; Figure S11: Top ten residues from time-resolved force distribution analysis (TRFDA); Figure S12: Structural alignment of PICK1 PDZ, PTP-BL PDZ2, and PSD-95 PDZ3.
