# Supplementary material for: Three Binding Conformations of BIO124 in the Pocket of the PICK1 PDZ Domain
Source: Cells. 2022 Aug 7;11(15):2451. doi: 10.3390/cells11152451 (PMC9368557; doi:10.3390/cells11152451)
Supplement: Supplementary file 1 [file cells-11-02451-s001.zip › cells-1836099-supplementary.pdf]

cells

Article

Three Binding Conformations of BIO124 in the Pocket of the PICK1 PDZ Domain

Amy O. Stevens [1], Samuel Luo [2] and Yi He [1,3,*]

1 Department of Chemistry and Chemical Biology, University of New Mexico, Albuquerque, NM 87131, USA
2 Albuquerque Academy, Albuquerque, NM 87131, USA
3 Translational Informatics Division, Department of Internal Medicine, University of New Mexico, Albuquerque, NM 87131, USA
* Correspondence: yihe@unm.edu

Supplemental Material:

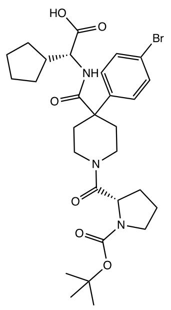

Figure S1. Structure of BIO124. .

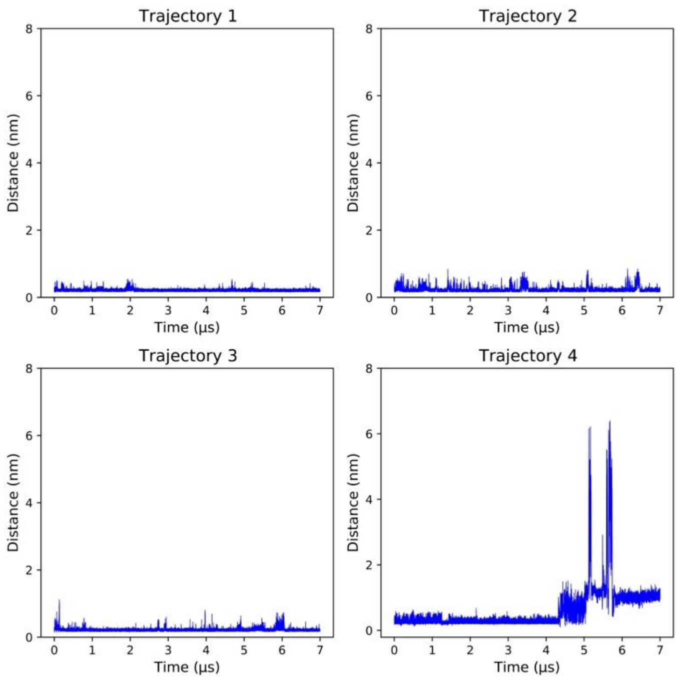

Figure S2. Distance between I37 of the PICK1 PDZ domain and BIO124. Spikes in distance after 4 us in trajectory 4 represent ligand dissociation from the binding pocket.

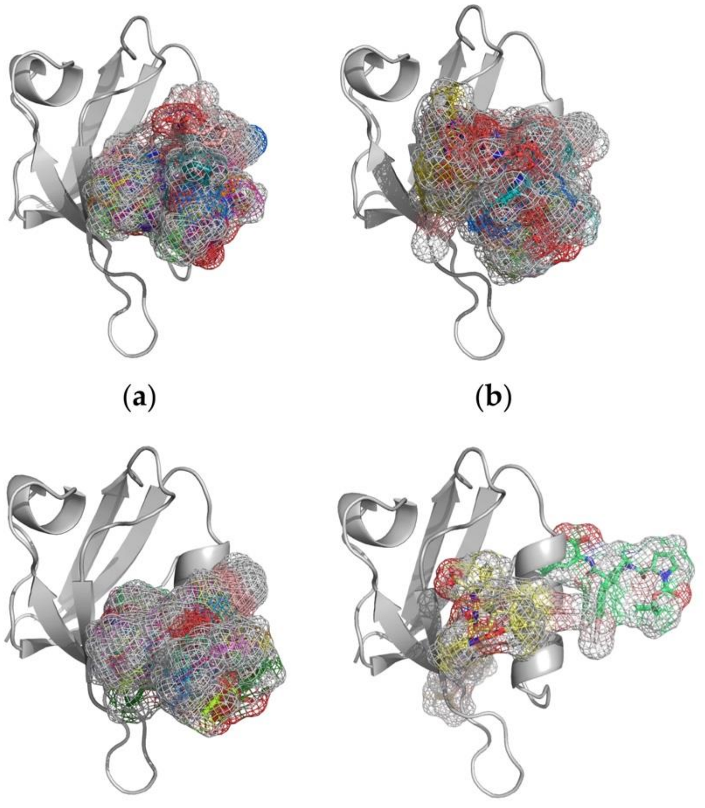

(**a**) (**b**)

(**c**) (**d**)

Figure S3. Cluster analysis of BIO124 per trajectory. (**a**) Trajectory 1. The top ten clusters shown represent 100% of the total frames. (**b**) Trajectory 2. The top ten clusters shown represent 100% of the total frames. (**c**) Trajectory 3. The top ten clusters shown represent 100% of the total frames. (**d**) Trajectory 4. The top three clusters shown represent 98.45% of the total frames. In two of the three clusters, the ligand has shifted from the binding pocket.

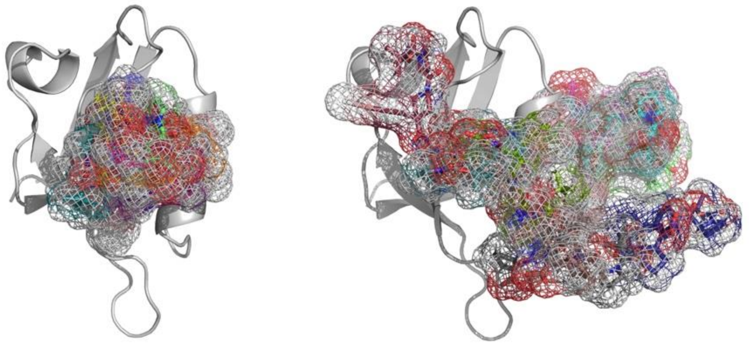

(**a**) (**b**)

Figure S4. Cluster analysis of BIO124 in trajectory 4. (**a**) The ten most probable positions of BIO124 during the first 4 us of trajectory 4. The clusters represent 100% of the total frames. (**b**) The ten most

probable positions of BIO124 during the last 3 us of trajectory 4. The clusters represent 100% of the total frames.

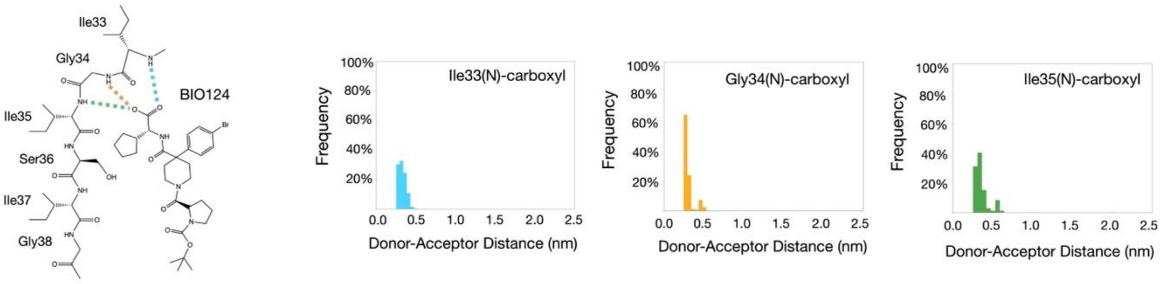

(**a**) (**b**)

Figure S5. State 0 hydrogen bonding network between BIO124 and the PICK1 PDZ domain. (**a**) Hydrogen bonds include I33-carboxyl (cyan), G34-carboxyl (orange), and I35 carboxyl (green). (**b**) Distance distribution between each hydrogen bonding pair in the complex. Note that each hydrogen bonding pair produces a Gaussian distribution.

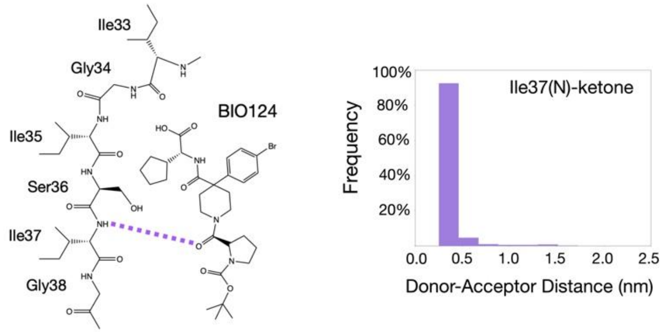

(**a**) (**b**)

Figure S6. State 1 hydrogen bonding network between BIO124 and the PICK1 PDZ domain. (**a**) Hydrogen bonds include I37-ketone (purple). (**b**) Distance distribution of the I37-ketone hydrogen bonding pair. Note the Gaussian distribution.

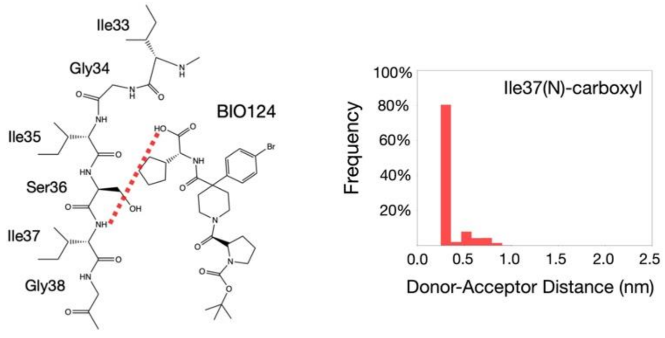

(**a**) (**b**)

Figure S7. State 2 hydrogen bonding network between BIO124 and the PICK1 PDZ domain. (**a**) Hydrogen bonds include I37-carboxyl (red). (**b**) Distance distribution of the I37-carboyxl hydrogen bonding pair. Note the Gaussian distribution.

While our MD simulations are performed at room temperature (293 K or ~20°C), the X-ray structure of the PICK1 PDZ-BIO124 complex was crystalized at 4°C (~277 K).[1] With this, we performed 12 replicates of 500 ns simulations at 277 K. Because all conformational shifts observed in our original simulations happened within the first 20 ns, we have assumed any conformational shifts will be observed by 500 ns in the new 277 K simulations. In total, we performed 6.0 µs of 277 K simulations.

We performed hydrogen bond analysis over all combined 277 K trajectories. In all combined 277 K trajectories, we identified the presence of three hydrogen bonding pairs between BIO124 and the PICK1 PDZ domain: I33-carboxyl, G34-carboxyl, and I35-carboxyl (Figure S8a). These pairs are in good agreement with the X-ray structure and state 0 observed in our original simulations. Notably, hydrogen bonding pairs corresponding to states 1 and 2 (I37-carboxyl and I37-ketone) are not identified in the 277 K simulations. Next, we performed a statistical analysis to rank the probability of each pair forming in the binding pocket (Figure S8b). The distance distributions for each pair produces a Gaussian distribution centered below 0.5 nm. These results indicate a single binding conformation with three stable hydrogen bonds that agree with the X-ray structure.

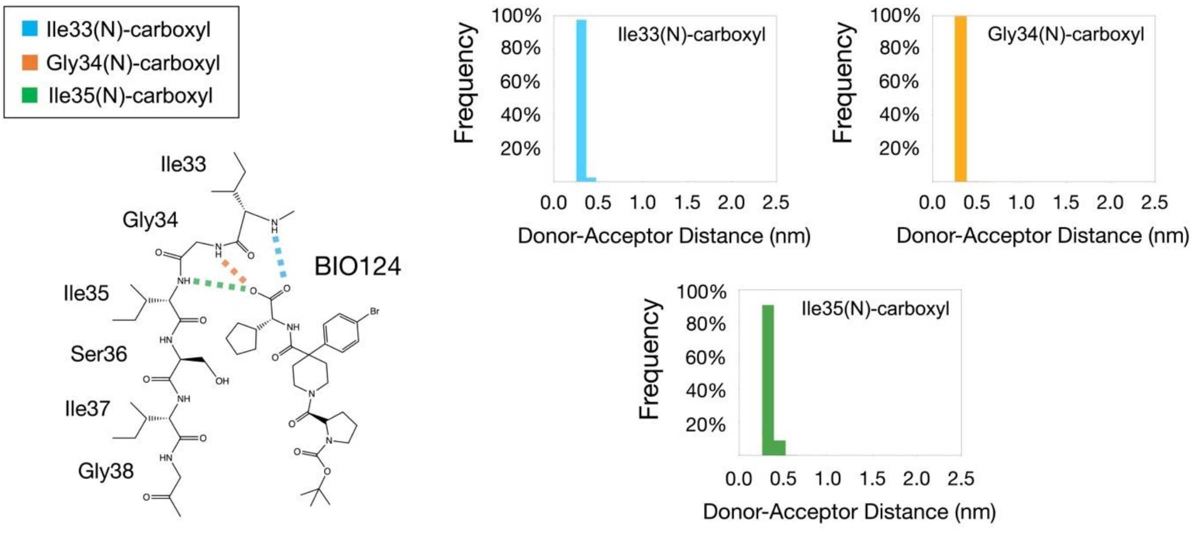

(**a**) (**b**)

Figure S8. 277 K hydrogen bonding network. (**a**) Hydrogen bonds include I33-carboxyl (cyan), G34-carboxyl (orange), and I35 carboxyl (green). (**b**) Distance distribution between each hydrogen bonding pair in the complex.

We calculated the atom-atom distance between each hydrogen bonding pair observed in the 277 K simulations (I33-carboxyl, G34-carboxyl, and I35-carboxyl) as well as the atom-atom distance between the additional hydrogen bonding pairs observed in states 1 and 2 in the original simulations (I37-carboxyl and I37-ketone). As shown in Figure S9, all trajectories keep the conformation of the starting structure with three hydrogen bonds formed throughout the simulations; the distances between I33-carboxyl (cyan), G34-carboxyl (orange), and I35-carboxyl (green) are below 0.5 nm in each trajectory. Oppositely, none of the trajectories observe hydrogen bonding between I37-carboxyl or I37-ketone; the distances between I37-carboxyl (red) and I37-ketone (purple) are greater than 0.5 nm

in each trajectory. This further indicates that state 1 and 2 are not present in the simulations at 277 K. The additional states observed in our original simulations may be a result of an increase in temperature that permits the sampling of states.

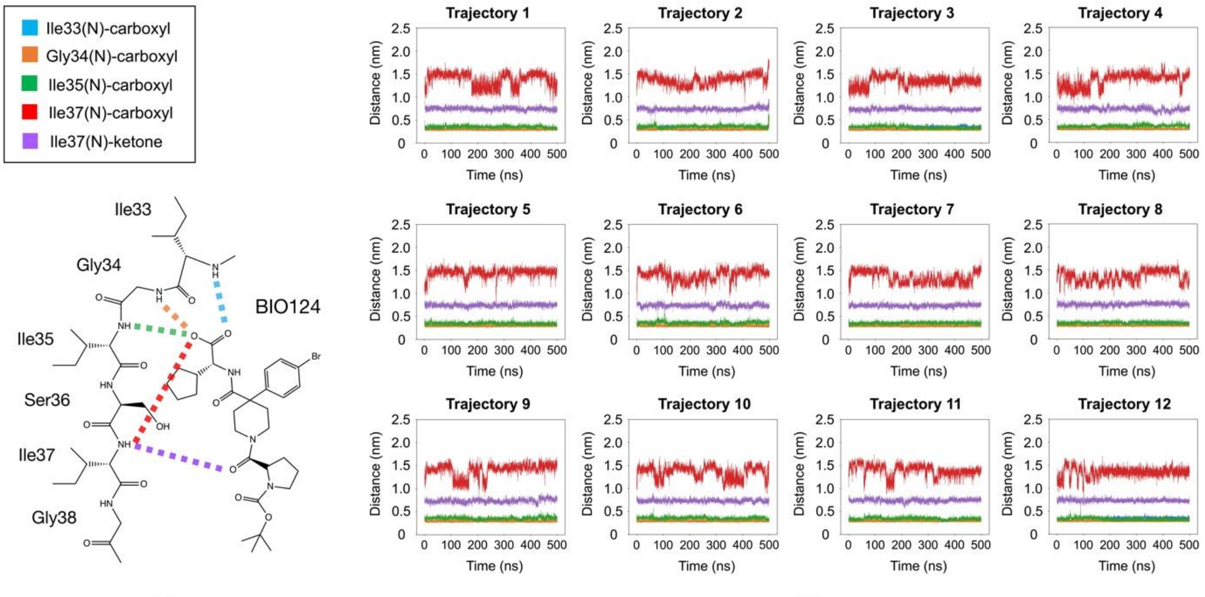

(a) (b)

Figure S9. 277 K atom-atom distance analysis of the hydrogen bonding pairs.

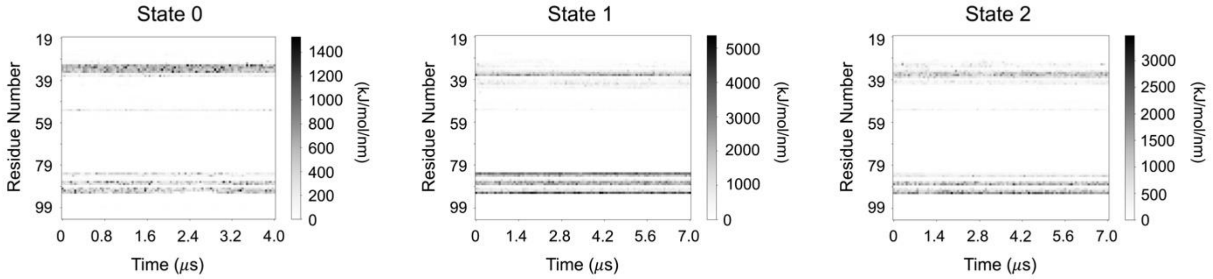

Figure S10. Summed time-resolved force distribution analysis (TRFDA) for each state of BIO124.

State 0		State 1		State 2	
Ile33	βB strand	Glu92	αB helix	Glu92	αB helix
Leu32	βB strand	Lys83	αB helix	Lys88	αB helix
Ile35	βB strand	Ile37	βB strand	Ile37	βB strand
Gly34	βB strand	Ala87	αB helix	Ser36	βB strand
Gln91	αB helix	Lys88	αB helix	Gln91	αB helix
Ala87	αB helix	Val84	αB helix	Ala87	αB helix
Ile90	αB helix	Ser36	βB strand	Gly38	βB strand
Glu92	αB helix	Gln91	αB helix	Val84	αB helix
Lys88	αB helix	Ile90	αB helix	Ile90	αB helix
Lys83	αB helix	Val86	αB helix	Ile35	βB strand

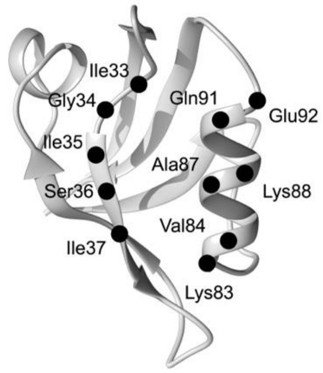

Figure S11. Top ten residues from time-resolved force distribution analysis (TRFDA). TRFDA reveals the top ten PICK1 PDZ residues with the greatest punctual stress across each state. In state 0, BIO124 induces the greatest punctual stress on the residues composing the βB strand. Oppositely in state 1 and state 2, BIO124 induces the greatest punctual stress on residues composing the αB helix.

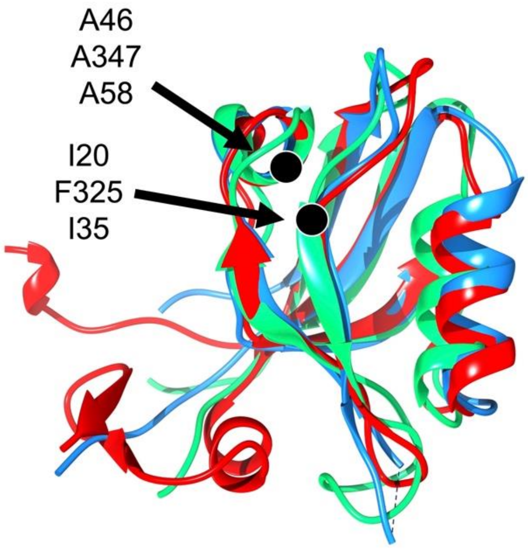

Figure S12. Structural alignment of the PICK1 PDZ domain (PDB ID: 2GZV, blue), PTP-BL PDZ2 (PDB ID: 3PDZ, green) and PSD-95 PDZ3 (PDB ID: 1BE9, red). A46 (PTP-BL PDZ2), A347 (PSD-95 PDZ3), and A58 (PICK1 PDZ) have been identified as allosteric residues in the αA helix. I20 (PTP-BL PDZ2) and F325 (PSD-95 PDZ3) have been identified as key residues in propagating signal to the αA helix. The structural equivalent of I20 and F325 in the PICK1 PDZ domain is I35.

References:

1. Pan, L.; Wu, H.; Shen, C.; Shi, Y.; Jin, W.; Xia, J.; Zhang, M. Clustering and synaptic targeting of PICK1 requires direct interaction between the PDZ domain and lipid membranes. *EMBO J.* **2007**, *26*, 4576–4587, doi:10.1038/sj.emboj.7601860.
